# Implementation of surveillance of invasive mosquitoes in Belgium according to the ECDC guidelines

**DOI:** 10.1186/1756-3305-7-201

**Published:** 2014-04-26

**Authors:** Isra Deblauwe, Charlotte Sohier, Francis Schaffner, Laurence Marrama Rakotoarivony, Marc Coosemans

**Affiliations:** 1 Medical Entomology Unit, Department of Biomedical Sciences, Institute of Tropical Medicine (ITM), Nationalestraat 155, 2000 Antwerp, Belgium; 2 Avia-GIS, Agro-Veterinary Information and Analysis, Risschotlei 33, 2980 Zoersel, Belgium; 3 National Centre for Vector Entomology, Institute of Parasitology, University of Zürich, Winterthurerstrasse 266a, CH-8057 Zürich, Switzerland; 4 European Centre for Disease Prevention and Control (ECDC), Tomtebodavägen 11a, 171 83 Stockholm, Sweden; 5 Department of Biomedical Sciences, Faculty of Pharmaceutical, Veterinary and Biomedical Sciences, University of Antwerp, Universiteitsplein 1, B-2610 Antwerp (Wilrijk), Belgium

**Keywords:** Invasive mosquito species, ECDC guidelines, Points of entry, Colonised area, *Aedes*, Oviposition trap, MMLP trap, Larval sampling, Surveillance, Europe

## Abstract

**Background:**

In 2012, the new guidelines for the surveillance of IMS in Europe, produced by the European Centre for Disease Prevention and Control (ECDC), were tested in Belgium. This study aimed at (1) testing the usefulness and applicability in the field of the ECDC guidelines for the surveillance of IMS in Europe and (2) surveying IMS throughout Belgium.

**Methods:**

First, the scenarios, which Belgium is facing, were identified according to the ECDC guidelines. Second, the surveillance strategy and the methods were identified based on the guidelines and adjusted to the Belgium context. Two areas colonised by IMS and 20 potential points of entry (PoE) were selected. Mosquito Magnet Liberty Plus (CO_2_-baited) traps (23) and oviposition traps (147) were set-up, and larval sampling was performed monthly or bi-monthly from July till October 2012. Finally, the costs and workload of the surveillance activities were compared to the estimates provided by the ECDC guidelines.

**Results:**

Surveillance at 20 potential PoE (complying with scenario 1) revealed that no new IMS were established in Belgium. Surveillance at two sites colonised by IMS (scenario 2) indicated that although control measures have drastically reduced the *Ae. j. japonicus* population this species is still present. Furthermore, *Ae. koreicus* is permanently established. For both scenarios, the problems encountered are discussed and recommendations are given. In addition, the actual workload was lower than the estimated workload, while the actual costs were higher than the estimated ones.

**Conclusions:**

The ECDC guidelines are helpful, applicable and efficient to implement surveillance of IMS in Belgium. Recommendations were customised to the local context (political demands, salary and investment costs, and existing expertise). The workload and costs related to the preparatory phase (i.e., planning, contacts with the PoE, writing a protocol) were found to be missing in the cost evaluation suggested in the guidelines. Updates on the occurrence of IMS in Belgium and the related risk for disease agents they can transmit will only be available once a structured and permanent surveillance system is implemented.

## Background

The incidence and geographical spread of mosquito-borne diseases (MBD) is increasing in Europe [1], as demonstrated by the recent autochthonous outbreaks of dengue, chikungunya, West Nile and Usutu virus in humans and/or animals [2-5]. Increasing globalisation (global movement of goods, animals and humans), climate and environmental changes seem to be facilitating factors for these epidemics [6]. Introduction of the mosquito vectors and the pathogens they transmit through global transport is becoming a topical issue that cannot be ignored anymore.

Container-breeding mosquitoes of the *Aedes* genus (Culicidae), which are (potential) vectors of several arboviruses [7], have an invasive potential as their eggs can withstand desiccation for many months and thus survive a long transportation time [8]. Six species have already been introduced into Europe, of which at least four (*Aedes albopictus*, *Ae. aegypti*, *Ae. japonicus japonicus* and *Aedes koreicus*) became regionally established [7,9]. Since 2005, regular introductions of *Aedes* species through lucky bamboo and second hand tyre importation were observed in the Netherlands [10,11]. In 2000, one larva and one pupa of *Ae. albopictus* were collected on the premises of a tyre company in Belgium (East Flanders) [12] but the species did not survive in the area. This species was, however, reintroduced in 2013 at the same location [13]. During a mosquito inventory of Belgium (MODIRISK 2007–2010), two other established invasive mosquito species (IMS) were detected [14]. At one site (Natoye), *Ae. j. japonicus*, already found in 2002, seemed to be well established in 2008 without spreading to the surroundings [15]. At the other site (Maasmechelen), *Ae. koreicus* was found for the first time in 2008 and seemed only locally established [16]. Whereas *Ae. j. japonicus* most likely had been introduced through the second hand tyre trade, the introduction pathway of *Ae. koreicus* remains unclear as it was found in a forest near an industrial zone, without an evident link with a commerce route [16].

The risk for establishment of the most invasive mosquito *Ae. albopictus* in northern Europe is increasing [17,18]. Climatic conditions have become warmer and wetter in north-western Europe, and thus are more suitable for *Ae. albopictus*[17]. Furthermore, the number of imported chikungunya and dengue cases in Belgium is also increasing [19,20]. The fact that IMS are able to enter Belgium and to establish, together with the global emergence of MBD emphasizes the need for the implementation of IMS surveillance in Belgium to detect possible foci of introduction and establishment at an early stage. Early detection of IMS allows appropriate and rapid implementation of control measures, and thus contributes to prevent MBD; while the surveillance of their abundance and further spread in colonised areas is needed for timely risk assessments of pathogen transmission to humans or animals [21,22].

Besides the public health concern, epidemics of MBD can have a considerable economic impact. For example, the medical costs of the chikungunya outbreak on La Réunion was 43.9 million euros [23]. The probability for early detection of an IMS or MBD, or for rapid interruption of transmission once an outbreak occurs, is directly related to an adequate surveillance system [24]. Only a few European countries have an active national IMS surveillance system (France, UK, the Netherlands, Germany) [25]. Although the interest in and need for IMS surveillance is increasing in European countries, the recent economic crisis makes it difficult to get the necessary funding [26]. An estimate of the full costs of a complete mosquito surveillance programme, is a preliminary requirement.

The European Centre for Disease Prevention and Control (ECDC) has produced guidelines to support the implementation of tailored surveillance of invasive mosquito vectors in Europe [27,28]. In March 2012, ECDC launched a call for candidature to evaluate these new guidelines in the field (pilot project). The Belgium candidature was accepted and IMS surveillance implemented during a six month project (ExoSurv). This study aimed at (1) evaluating the usefulness and applicability in the field of the ECDC guidelines for the surveillance of IMS and (2) surveying IMS throughout Belgium in the summer of 2012. The identification process of surveillance strategies, interpretation and adaptation of the methodology proposed in the guidelines to the Belgium context and constraints, as well as a comparison of the estimated and actual cost and workload were performed and described by the ITM staff only. ECDC coordinated the ECDC pilot project and VBORNET (network of medical entomologists and public health experts, funded by ECDC) provided technical support during the implementation. Thus, recommendations and evaluations are performed by independent bodies. The main results of the surveillance, at potential points of entry (PoE) and at two sites where IMS are known to be present, are given and discussed. Detailed results of the surveillance in terms of mosquito findings and efficacy of control methods can be found in an online available report [29].

## Methods

### Decision making process: development of a surveillance strategy using the ECDC guidelines, adaptation to the local situation in Belgium

In the ECDC guidelines [28], three scenarios are defined, of which two were identified in Belgium and used for the development of the surveillance strategy. Based on the recent Belgian mosquito inventory study MODIRISK [14], the whole country, except for two locations (see below), fulfils the criteria of scenario 1 of the guidelines, i.e. “no established IMS, but with risk of introduction and establishment”. The two remaining locations (Natoye and Maasmechelen), where two IMS are locally established, correspond to scenario 2 of the guidelines, i.e. “locally established IMS with low risk of spreading into new areas”. During the ExoSurv project key procedures were implemented following figure seven of the guidelines [28]. For scenario 1, the objective is the surveillance of potential PoE for the presence of IMS. For scenario 2, an additional objective is the surveillance (i) at colonised areas for presence and persistence and, (ii) at surroundings for spread.

### Operational process: identification of the sites at risk for introduction or spreading of IMS

**Figure 1:**
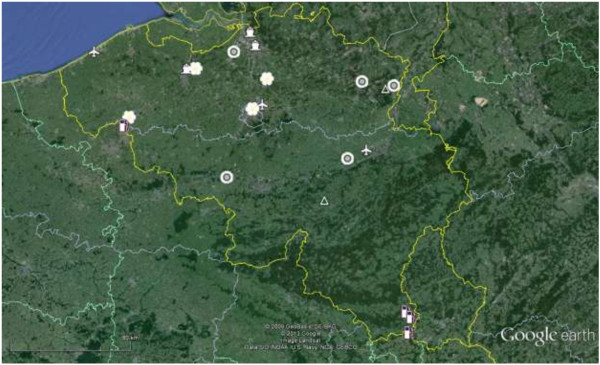
**Localisation of the different points of entry (PoE) and the two areas colonised by invasive mosquito species (IMS).** Symbols: airplane = airport; boat = port; flower = shelter or greenhouse for imported cutting plants; fruits or vegetables; tyre = platform of imported used tyres; petrol pump = main parking lot near highway at country border; triangle = colonised area.

For scenario 1, PoE included platforms of imported used tyres, shelters/greenhouses for imported cutting plants (e.g. lucky bamboo), main parking lots near highways at country borders and near road axes connected to IMS-colonised areas, ports and airports [28]. Based on the evaluation of the risk to introduce IMS according to the ECDC guidelines, we selected nine sites already sampled during the MODIRISK project [14] and 17 additional PoE. A standard email was sent or a phone call was made to request permission to survey each of the 26 selected PoE. A limited number of new sites (n = 7) were visited in advance to check the suitability for import of IMS and the willingness of the landowners to collaborate with the study team. For scenario 2, the two colonised areas were located at the imported used tyre company in Natoye and at the industrial zone ‘Op de Berg’ in Maasmechelen (Figure 1, Additional file 1). Both had already been surveyed during the MODIRISK project [14].

### Operational process: collection methods

Table 1 compares methods used in this ExoSurv project and methods recommended by the ECDC guidelines. All six *Aedes* species introduced in Europe (*Ae. albopictus*, *Ae. aegypti*, *Ae. j. japonicus*, *Ae. koreicus*, *Ae. atropalpus* and *Ae. triseriatus*) were targeted. During the six-month project there were four months of actual surveillance (July till October 2012). The same sampling effort was applied in all PoE to obtain comparable data. The Mosquito Magnet Liberty Plus (MMLP, CO_2_-baited, Woodstream Corporation, Lititz, PA, USA) trap was used at all PoE and two colonised areas (Natoye and Maasmechelen) as it has at least a fair efficacy for all invasive *Aedes* species (see table two in guidelines [28]) and it can be run for a week without additional power supply. Moreover, MMLP traps were more efficient than the BG-Sentinel traps in capturing *Ae. koreicus* and in Belgium this trap scored for the highest diversity and quantities of mosquitoes [16,25]. For *Ae. albopictus* commercial CO_2_-baited traps (e.g., Mosquito Magnet Liberty, MML) were advised for routine surveillance in North Central Florida [30]. Sampling with the MMLP trap was carried out during one week per month. At least three oviposition traps (small black plastic bucket, 2/3 filled with an oak infusion and a piece of polystyrene as oviposition support) were set up at each PoE as recommended in annex three of the ECDC guidelines [28]. At the two colonised IMS sites, oviposition traps were set-up in groups of two per subsite. Subsites are extra sampling sites within a 5 km perimeter around the colonised site and were selected at 1, 3 and/or 5 km in south, southwest, southeast, east, west, north, northwest and northeast direction from the original introduction point [29]. The larvicide VectoMax® (Sumitomo Chemicals) was added to the ovitraps at the colonised sites to prevent proliferation of the IMS (starting in August). All ovitraps were run permanently and sampled every three to four weeks. The placement of both trap types was done according to the ECDC guidelines (annex three) [28], avoiding open terrains, protected from wind, out of sight, in dense shrubs, and fully labelled. At each PoE, 20 vessels, if present, were checked for the presence of mosquito larvae. At used tyre companies, larval searches were carried out each month, and every two months at the other PoE. At and around the IMS-colonised sites, 40 to 130 vessels, depending on the available type of potential breeding site (tyres or other containers), were checked for larvae each month. A potential breeding site is a single vessel or a group of the same vessels (e.g. a stock of tyres, lucky bamboo containers in the same shelter) in which mosquito larvae can develop.

**Table 1:** Comparison of the recommended (ECDC) and implemented (ExoSurv) mosquito collection methods at the points of entry (PoE) (Scenario 1) and the areas colonised by invasive mosquito species (IMS) (Scenario 2)

**A. PoE (Scenario 1)**						
**Type of PoE**	**Methods and traps**	**ECDC**		**ExoSurv**^**2**^		
		**Density traps**	**Frequency**	**Density traps**		**Frequency**
**Storage sites for imported used tyres *Key procedure***	**BG/MMLP**^**1**^	1/5000 m^2^	2/month	1.3/5000 m^2^		1/month
	**LS**^**1**^	1/10 tyres	2/year	20 tyres		1/month
	**OT**^**1**^	0	NA*	3.8/5000 m^2^		1/month
**Shelters/greenhouses for imported plants/fruits/vegetables *Key procedure***	**BG/MMLP**	1/5000 m^2^	2/month	0.4/5000 m^2^		1/month
	**LS**	20 vessels	2/year	20 vessels		2/year^†^
	**OT**	0	NA	1.1/5000 m^2^		1/month
**Parking lots at country borders *Key procedure***	**BG/MMLP**	0	NA	0.2/5000 m^2^		1/month
	**LS**	10 vessels	2/year	20 vessels		2/year^†^
	**OT**	1/2500 m^2^	2/month	0.2/2500 m^2^		1/month
**Ports *Key procedure***	**BG/MMLP**	0	NA	0.1/5000 m^2^		1/month
	**LS**	0	NA	20 vessels		2/year^†^
	**OT**	1/5000 m^2^	2/month	0.3/5000 m^2^		1/month
**Airports *Optional procedure***	**BG/MMLP**	1/2.5 ha	2/month	0.1/2.5 ha		1/month
	**LS**	0	NA	20 vessels		2/year^†^
	**OT**	1/1 ha	1/month	0.1/1 ha		1/month
**B. IMS-colonised areas (Scenario 2)**						
**Surveillance measures**	**Methods and traps**	**ECDC**		**ExoSurv**		
		**Density traps**	**Frequency**	**Density traps**		**Frequency**
				**Maasmechelen**	**Natoye**	
**Inspection of colonised area *Key procedure***	**BG/MMLP**	1/20 ha	2/month	0.1/20 ha	0.3/20 ha	1 or 4/month
	**LS**	40 vessels	2/month	17 vessels	40-60 vessels	1/month
	**OT**	1/5 ha	2/month	0.2/5 ha	0.6/5 ha	1/month
**Quality & efficacy of control *Key procedure***	**BG/MMLP**	4/site	B&A appl^††^	0	1/site	1/month
	**LS**	0	NA	0	40-60 vessels	1/month
	**OT**	20/site	B&A appl	0	6/site	1/month
**Inspection around colonised area *Key procedure***	**BG/MMLP**	0	NA	0	0	NA
	**LS**	0	NA	23 vessels	25-70 vessels	1/month
	**OT**	1/15 ha	1/month	0.09/15 ha	0.08/15 ha	1/month
**Quality & efficacy of control *Not recommended***	**BG/MMLP**	4/site	B&A appl	0	0	1/month
	**LS**	0	NA	0	20-40 vessels	1/month
	**OT**	20/site	B&A appl	0	2/site	1/month

^1^BG = BG-Sentinel trap, MMLP = Mosquito Magnet Liberty Plus (CO_2_-baited) trap, LS = larval sampling, OT = oviposition traps.

^2^Period of sampling: July - October 2012; only MMLP traps were used and human landing collection was not performed due to the high workload and the strict time schedule.

*NA = not applicable.

^†^in August and September 2012.

^††^B&A appl = before and after applications.

Mosquito adults and larvae were identified using digital and dichotomous keys [28,31,32], reference material and a specific description of *Ae. koreicus*[25]. When eggs from oviposition traps did not hatch in the laboratory, they were identified using MALDI-TOF mass spectrometry by a private company (Mabritec, Riehen, Switzerland) as described [33-35].

### Technical training and support

During the operational process training and support was provided by VBORNET [36]. In the preparatory phase, support was given in the selection of the PoE. Two days were spent in the field to advise on the placement of traps and to help in the recognition of mosquito larval habitats. One day was spent in the laboratory for morphological identification training. At the end of the programme a quality check of identifications was performed by one of the authors (FS).

### Data management and analyses

The Smart-To-Web tool Vecmap [37] was used to enter the data in the field. All variables are presented in the Microsoft Access entity relationship diagram (Additional file 2). Most variables proposed in the guidelines were used. Nomenclature of Territorial Units for Statistics (NUTS) and altitude, mandatory fields in the guidelines, were not used because of the small scale of spread at the IMS-colonised sites. Identification with MALDI-TOF mass spectrometry was indicated in the “comments” of the database (Additional file 2).

The indicator “species richness”, defined as the number of species in a definite sample unit, was used to present surveillance data.

### Calculation of the estimated and actual costs and workload

Estimated costs and workload (1 working day (wd) = 7.5 h) were calculated using table three, ten and eleven of the ECDC guidelines [28] and taking into account the actual surveillance period (four months), frequency (1/month), the number of sites or km^2^ and the number of visits and sites visited per day. The mean distance between the PoE and the total distance covered at IMS-colonised areas was calculated using Google Maps. The total distance covered at the colonised areas is an estimate as the travel route could change from visit to visit. The different procedures at the colonised areas were grouped as they were done during the same visits. Only trap density was not adjusted because of the variations in trap number/vessel number to be checked at the different PoE and the two colonised sites (Table 1).

To calculate actual costs and workload, all expenses and working hours were registered during the seven month period of the project that included the four month field work. Working hours were entered in a time registration software and grouped in different categories according to the guidelines (field and laboratory investigations, data processing and communication/dissemination). Workload was divided between scenario 1 and 2 based on the proportion of days in the field (field investigations), the proportion of tubes containing adult or larval mosquito samples and of polystyrene pieces of the ovitraps checked in the laboratory (laboratory investigations), and the proportion of time spent on data processing, communication and dissemination.

## Results and discussion

### Scenario 1: surveillance at potential PoE in Belgium

Two used tyre companies (one MODIRISK site) and one parking lot were not accessible, one shelter for imported plants went bankrupt, one parking lot could not be contacted and one port was found to be at low risk for IMS import during a visit (dry goods, few import). Finally a total of 20 of the 26 selected potential PoE were retained, including five storage sites for imported used tyres, five shelters/greenhouses for plants (or fruits and vegetables), four parking lots at country borders, three ports and three airports (Figure 1, Additional file 1). The advised density of traps and sampling frequency at the PoE was reduced according to the available budget (€ 82,495) and traps (25 MMLP traps, 17 oviposition traps, 7 BG-Sentinel traps, 6 gravid traps) (Table 1). At used tyre platforms, larval search of 1/10 tyres (i.e. between 20 and 200 tyres per company) was too intensive for one person taking into account the strict time schedule to be followed. Instead, at least 20 used tyres were checked for larvae per company during the present survey.

A summary of the number of samplings, total specimens and species number per collection method for each PoE type is presented in Table 2. The Additional file 3 provides the results by species. The number of mosquito species and adults captured with the MMLP was highest at the storage sites for imported used tyres (a total of 11 species and 357 mosquitoes), but no IMS were captured with the MMLP traps in the 20 PoE (Additional file 3). The placement of the MMLP trap, inside or outside the building, seems to influence the number of adults collected. The traps were placed as close as possible to the location where cargo is unloaded or opened, which is often inside the building. The observed number of species (n = 14) caught with MMLP traps seemed to be influenced by the presence of mosquito larval breeding sites at the PoE (artificial containers, e.g. tyres) and nearby them (natural areas, e.g. ponds or forests). During the MODIRISK project, 12 mosquito species were captured with MMLP traps in 26 PoE [14], of which eight areas were revisited during this study. Although the mean number of adult mosquitoes per trap (n = 12) was lower than during the MODIRISK project (n = 38), species richness was higher during the ExoSurv project (ExoSurv = 14 species, MODIRISK = 12 species). The abnormal dry period between August and September 2012 [29,38] might explain the low number of 780 mosquitoes sampled. The higher species richness is probably the effect of the greater number of trap weeks and the inclusion of new PoE. Ten of the 12 species collected during the MODIRISK project were also collected during ExoSurv (Additional file 3), indicating that the results are coherent. At all storage sites of imported used tyres mosquito larvae (a total of 602 larvae) were found, but no IMS (Additional file 3). At the other PoE only a few vessels (28 vessels of 19 potential breeding sites) were found and no mosquito larvae were collected. The search for larval breeding sites is of course a learning process, which improved towards the end of the Exosurv project. No exotic *Aedes* eggs were collected with the ovitraps. Only *Ae. geniculatus* eggs, identified with MALDI-TOF MS, were collected once at a storage site for imported used tyres. Thus, in 2012 the risk for public or animal health seemed very low, as *Ae. albopictus* was not detected during the survey. However, it is difficult at this point to really evaluate in Belgium the specificity and sensitivity of the surveillance methods advised in the guidelines. During recent surveillance activities in 2013 in Belgium, *Ae. albopictus* was caught with a MMLP trap used during another study at a platform for imported used tyres [13], while our MMLP at the same site did not catch it. Mosquito density was probably still too low to be captured by both traps. However, in neighbouring countries similar surveillance activities have proven to be able to detect early introduction of IMS [11,39,40].

**Table 2:** **Number of samplings, total specimens, species number or positive samplings per collection method at each type of point of entry (PoE) (Scenario 1) and at the invasive mosquito species (IMS)-colonised areas Natoye ( *Aedes j. japonicus *) and Maasmechelen ( *Ae. koreicus *) (Scenario 2) (period of sampling: July - October 2012)**

**PoE (Scenario 1)**		**Storage sites for imported used tyres**	**Shelters/greenhouses for imported plants/fruits/vegetables**	**Parking lots at the country border**	**Ports**	**Airports**
**Number of sites**		**5**	**5**	**4**	**3**	**3**
**MMLP**^**1**^	**N° trap weeks**^**2**^	19/19	16/19	8/14	10/12	11/11
	**Total specimens**	357	161	38	93	131
	**Species richness**	11	4	8	6	3
**OT**^**1**^	**N° samplings**^**3**^	36/46	40/45	35/37	26/27	27/28
	**Presence IMS eggs**	no	no	no	no	no
**LS**^**1**^	**N° samplings**^**4**^	18 (7 PBS^1^)	9 (7 PBS)	5 (4 PBS)	3 (3 PBS)	7 (5 PBS)
	**Total specimens**	602	0	0	0	0
	**Species richness**	5	0	0	0	0
**IMS-colonised areas (Scenario 2)**		**Natoye**^**5**^		**Maasmechelen**^**6**^		
		**At colonised area**	**Around colonised area**	**At colonised area**	**Around colonised area**	
**MMLP**	**N° trap weeks**	5/5	NA	M1: 14/16 & M2: 2/8	NA	
	**Total IMS specimens**	0	NA	M1: 7 & M2: 1	NA	
**OT**	**N° samplings**	36/40	116/132	72/73	81/84	
	**N° samplings with IMS eggs**	5/36	6/116	0/72	0/81	
**LS**	**N° samplings**	14 (3 PBS)	69 (18 PBS)	58 (17 PBS)	52 (23 PBS)	
	**Total IMS specimens**	74	13	250	0	

^1^MMLP = Mosquito Magnet Liberty Plus (CO_2_) trap, OT = oviposition trap, LS = larval sampling, PBS = potential breeding site (=a single vessel or a group of the same vessels (e.g. a stock of tyres, lucky bamboo containers in the same shelter) in which mosquito larvae can develop).

^2^N° of effective MMLP trap weeks/n° trap weeks planned.

^3^N° samplings with polystyrene piece found back/n° samplings planned.

^4^N° samplings with number of PBS sampled between brackets (a stock of tyres is one PBS, but at least 20 tyres were checked for larvae during each sampling).

^5^the colonised area at Natoye is located within 500 m from the used tyre company.

^6^the colonised area at Maasmechelen is located within 1 km from the industrial area ‘Op de Berg’; two MMLP traps were set-up (M1 and M2).

It was not always possible to select the ideal location of the trap at PoE, especially at ports, airports and parking lots. At these PoE, the risk of damage to the trap or vandalism is greater than at other PoE (passing people or vehicles e.g. forklifts). Especially MMLP traps and ovitraps were prone to be stolen and vandalised. This was the case with MMLP traps at two parking lots, which is the main reason why they were not advised at this type of PoE in the ECDC guidelines. From September, MMLP traps were secured with chains and padlocks. Although the surveillance of airports is an optional procedure (Table 1), the three main cargo airports in Belgium were selected because of possible dissemination of exotic mosquitoes upon opening of containers.

During the Exosurv project, preparation time was too short to carry out a thorough risk assessment of the PoE as advised in the ECDC guidelines [28], and to get the necessary permissions in advance (e.g. at airports). An investigation based on interviews and questionnaires, as performed in the Netherlands [41], is necessary to devise a hierarchical list of PoE ranked from high risk to low risk [28]. This could be done by the research institution implemented in the surveillance. According to the resulting hierarchical list, the most important PoE to be surveyed can then be selected, taking into account the available personnel and budget. It was noted that no list of companies at risk for importing IMS are available in Belgium, especially companies importing used engine tyres, or lucky bamboo. Information on the existing import companies could be gathered from the Belgian custom services. Owing to the possible impact on public and animal health, countries should consider legislation with specific regulations (e.g. storing tyres out-of-water, changing and cleaning the recipients of lucky bamboo), which allows the inventory and regular visit of these companies.

Comparison of the actual and estimated (Additional file 4) workload for scenario 1 is presented in Table 3. The total actual workload for scenario 1 was lower compared to the estimated workload. In particular, the actual workload for field investigations was much lower (−60 wd), probably due to the fact that different types of PoE were visited on the same day. The number of working days by type of PoE varied from 3.6 (for shelters/greenhouses) to 8 days (airports) per site (Additional file 5). Furthermore, the actual workload was higher than the estimated workload for laboratory investigations (+13 wd), communication and dissemination (+18 wd) and data processing (+3 wd) (Table 3). The higher workload for laboratory investigation is explained by the identification learning process required at the beginning of the surveillance. The actual workload for communication and dissemination, essential for a good cooperation, was clearly underestimated. The relative workload for these items will probably decrease with longer surveillance periods, although refreshing courses and permanent communication remains an absolute priority.

**Table 3:** Comparison of the estimated workload, applying ECDC workload rates and formulas, and the actual workload of the ExoSurv project presented by category of personnel (workload in working days)

	**Scenario 1**		**Scenario 2**		**Scenario 1 + 2**			
	**ECDC**^**1**^	**ExoSurv**^**2**^	**ECDC**^**1**^	**ExoSurv**^**2**^	**ECDC**^**1**^	**ExoSurv**^**2**^		
						**Total**	**Post-doc**	**Technician**
**Field investigations**	101	41	20	41	121	82	24	58
**Laboratory investigations**^**3**^	13	26	67	22	80	48	35	13
**Data processing**	4	7	35	12	39	19	11	8
**Communication/dissemination**	4	22	31	44	35	66	41	25
**Total surveillance workload**	**122**	**96**	**153**	**119**	**275**	**215**	**111**	**104**
**Preparatory phase**		33		13		46	21	25
**Total workload**		**129**		**132**		**261**	**132**	**129**
**Available wd 2012**						242	121	121
**Extra wd 2012**						19	11	8

^1^Four months surveillance, including communication and dissemination, excluding preparatory phase (following scenario 1 & 2, without adjustment for trap density).

^2^One month preparation (Jun-Jul, preparatory phase), four months surveillance (Jul-Oct) and two months communication and dissemination (Nov-Dec).

^3^Workload for laboratory investigations was divided between scenario 1 & 2 based on the number of tubes with adults and larvae, and of polystyrene pieces checked (PoE = 54%, IMS-colonised areas = 46%).

Comparison of the actual and estimated (Additional file 4) costs for scenario 1 is presented in Table 4. In contrast to the workload, the total actual cost (€ 4,759) for scenario 1 was slightly higher than the estimated cost (€ 4,376). For field investigations, actual costs were almost the same as the estimated costs. The actual costs also included those of polystyrene, a mobile phone card and of propane tanks, CO_2_ cartridges, chains and padlocks for the MMLP traps. In addition, the kilometre rate was higher during this project (€ 3.4/km, including car rent and gasoline; € 0.3/km in ECDC guidelines). As with the workload, the opportunity to visit several PoE in one day and hence to decrease the number of kilometres was not taken into account in the estimates based on the guidelines (Additional file 5). Further, actual costs for laboratory investigations (+€ 229), communication and dissemination (+€ 166) were higher than the estimated costs. Actual costs for communication and dissemination included costs of printing and sending the report, and of train tickets for meetings. The actual costs for laboratory investigations included consumables (ethanol, plastic bags and boxes, silica gel, tubes and filters) for manipulation and storage of the mosquitoes, but not the molecular/MALDI-TOF MS identification.

**Table 4:** Comparison of the estimated working costs, applying ECDC cost rates and formulas, and the actual working costs of the ExoSurv project (costs in euro)

	**Scenario 1**		**Scenario 2**		**Scenario 1 + 2**		**Difference ExoSurv - ECDC (Scenario 1 + 2)**
	**ECDC**^**1,2**^	**ExoSurv**^**1**^	**ECDC**^**1,2**^	**ExoSurv**^**1**^	**ECDC**^**1,2**^	**ExoSurv**^**1**^	
**Field investigations**	4,297	4,294	1,848	3,777	6,145	8,071*	1,926
**Laboratory investigations**	59	288	163	146	222	434**	212
**Data processing**	9	0	51	69	60	69***	9
**Communication/dissemination**	11	177	33	177	44	354^†^	310
**Total surveillance cost**	**4,376**	**4,759**	**2,095**	**4,169**	**6,471**	**8,928**	**2,457**

^1^excluding investment (traps), training and salary costs, including travel and consumable costs for four months of actual surveillance.

^2^Following scenario 1 & 2, without adjustment for trap density, for four months of surveillance and excluding leaflet costs.

*Including costs of car rent, gasoline, mobile phone card, polystyrene and propane tanks, CO_2_ cartridges, chains and locks for Mosquito Magnet Liberty Plus traps.

**Including costs of ethanol, plastic bags and boxes, silica gel, tubes and filters, excluding costs for molecular/MALDI-TOF identification.

***Including costs of meteorological data from the Royal Meteorological Institute (RMI).

^†^Including costs of printing and sending report and of train tickets for meetings. No flyer was edited and printed, only the report for the policy makers was provided.

### Scenario 2: surveillance at two IMS-colonised sites in Belgium

The density of ovitraps actually placed around the IMS-colonised areas was 0.08 ovitraps/15 ha in Natoye and 0.09 ovitraps/15 ha in Maasmechelen as compared to the recommended density of 1 ovitrap/15 ha. Also, the frequency of visits was lower (once a month in the present study as compared to twice a month recommended by the guidelines). On the other hand, larval sampling around the colonised area was added, although not recommended in the guidelines (see Table 1) because of the need for expertise to identify suitable breeding places and high costs in terms of workload. However, during the present study, this method was found to be essential to estimate the present spread of these IMS and the efficacy of control measures [29]. Therefore, we suggest that the two variables ‘breeding site code’ and ‘larval habitat type’ are included in the database.

A summary of the number of samplings, total IMS specimens or positive IMS samplings per collection method for each IMS-colonised site is presented in Table 2.

At the colonised site of Natoye, ten years after the first detection of *Ae. j. japonicus*[15], control measures were first implemented in 2012. First of all mechanical treatment was carried out (e.g. storing tyres out-of-water), followed by larviciding with *Bacillus thuringiensis israelensis* (Bti) and with a mixture of Bti and *B. sphaericus* (respectively VectoBac® WG and VectoMax®, Sumitomo Chemicals) [29]. This drastically reduced its population but some specimens were still found throughout the season, with evidence of a limited spread outside the tyre company, mainly in the southwest direction, up to 2 km [29]. Although oviposition traps were efficient to check for the presence and spread of *Ae. j. japonicus* (11/152 ovitraps, Table 2), a problem with the ovitraps concerned the removal of the oviposition supports (polystyrene pieces) presumably by birds or rodents. For example, at and around the colonised area of Natoye, 6% and 10%, respectively, of the polystyrene pieces were lost, while 26% and 17% were found next to the trap. This trend increased with the use of the larvicide VectoMax® in the ovitraps, which might attract birds or rodents because of the formulation using granules of corn. Animals might also have searched for water during the dry summer and repeatedly visited the traps. A solution might be to attach the polystyrene piece to the vessel or add a mesh over the trap to avoid removal by animals. Polystyrene pieces were brought to the laboratory for egg hatching and larvae were further identified as *Ae. j. japonicus* (Table 2) and *Ae. geniculatus*. Unhatched eggs further analysed by MALDI-TOF MS were determined as *Ae. geniculatus*. These eggs were probably in diapause [42]. Although *Ae. j. japonicus* larvae were collected in tyres close to the MMLP trap, adults of this IMS were not collected with the MMLP trap, and other trapping methods might be more appropriate to collect this species (gravid traps [25,28], human landing collection [15]). In contrast to table two of the ECDC guidelines [28], human landing collection seems to have a much better efficacy to collect *Ae. j. japonicus* than the MMLP trap [15].

At the colonised site of Maasmechelen, five years after its first detection, *Ae. koreicus* was still established in an area limited to 3 km^2^ around the industrial zone only [29]. In contrast to *Ae. j. japonicus*, no *Ae. koreicus* eggs were collected with the oviposition traps at Maasmechelen. Ovitraps seemed less efficient than MMLP traps to detect *Ae. koreicus* in low density areas [29]. Also in 2009 only two of the 17 ovitraps were positive for *Ae. koreicus* in two of the seven months sampled [16]. It might be an option to replace the black plastic ovitraps with metal or light-coloured plastic ovitraps, as larvae were often collected in metal or light-coloured containers [29]. Another alternative is to use another infusion (grass leaves or from known breeding sites) to attract *Ae. koreicus*. For *Ae. koreicus*, little is known on the efficacy of the method of collection (see table two in the guidelines [28]). A previous study [25] indicates, however, that gravid traps and MMLP traps have a higher efficacy to collect *Ae. koreicus* than BG-Sentinel and oviposition traps.

As in scenario 1, the total actual workload was lower than the estimated workload (Additional file 4) for scenario 2 (Table 3), especially for laboratory investigations (−45 wd) and data processing (−23 wd). The much lower trap density (ovitraps and MMLP traps) partly explains the low actual workload for laboratory investigations. No complex analysis and mapping was carried out, which decreased the data processing workload. Instead, for field investigations (+21 wd) and for communication and dissemination (+13 wd), the actual workload was higher. Most of the time, two people went to the field for the scenario 2 investigations because of the number of ovitraps that required visiting and the larval sampling covering a surface of 19 km^2^ (there was no community participation). As in scenario 1, actual workload for communication and dissemination was partly higher due to the specificity of the pilot study, which involved a short surveillance period and an evaluation process. Still, no flyer needed to be edited and printed, which would increase the workload for communication and dissemination. It is clear that the workload will depend on the experience of a country and the demands of the policy makers for communication (e.g. editing a flyer).

In contrast to the workload, the actual costs were twice the estimated costs (Additional file 4) for scenario 2 (Table 4), especially the actual costs for field investigations. The higher km rate (€ 3.4/km, including car rent and gasoline) increased the actual cost. As in scenario 1, actual costs for communication and dissemination (+€ 144) were higher than the estimated costs. For data processing (+€ 18) and laboratory investigations (−€ 17), the difference was small, in contrast to the workload.

### Both scenarios: preparatory phase of surveillance implementation

For both scenarios the costs (investment, capacity building, etc.) and workload (protocol, site selection, preparation of material, etc.) related to the preparatory phase have to be estimated in the local context and taken into account before implementing the surveillance activities. These activities were not clearly identified in the cost estimations described in the guidelines. Total costs (including salaries) will be very different according to the country, but an example is given for Belgium. For the present study (ExoSurv), the price of 120 new oviposition traps (€ 302; € 2.52/trap) and two MMLP traps (€ 1,825; € 912.5/trap) was an investment cost, which was higher than the average cost provided in the guidelines, partly because prices depend on local rates. In addition, specification of degree and number of people required for each specific task and workload have to be estimated. In Belgium, one experienced post-doc and one technician performed the surveillance and the few, but very valuable days of training and support received (not included here in actual costs), were enough to continue the work. However, the need for training and support will increase when this competence is not available in a country, which will increase the costs for capacity building.

## Conclusions

No additional IMS were detected during the surveillance at 20 PoE in Belgium in 2012 (July till October). However, existence of trades posing a risk for introduction of IMS and of IMS colonies in neighbouring countries pleads for routine IMS inspections at high risk PoE to detect the presence of IMS as early as possible. In neighbouring countries awareness has risen during the last few years on the risk posed by exotic and indigenous vector species [11,43-45], although it has not always avoided MBD outbreaks (e.g. southern France) [2]. To deal with vectors and vector-related public and animal health issues, several countries have established a nationwide coordinating organisation or centre, e.g. the Centre for Monitoring of Vectors (CMV) in the Netherlands [46], the Centre National d’Expertise sur les Vecteurs (CNEV) in France [47] and the Medical Entomology and Zoonoses Ecology Group, Public Health England in the UK [48]. Only a structured and permanent surveillance system will assure a regular update of the occurrence of IMS in Belgium and the related risk of the diseases (e.g. West Nile, dengue, chikungunya) they can transmit. However, the role of native mosquito species as potential vectors of arboviruses (e.g. West Nile fever) should also not be neglected. Moreover, a structured and permanent surveillance system requires a high political commitment, which can only be achieved by appropriate advocacy.

The ECDC guidelines were very useful in the set-up of a surveillance study in Belgium, particularly for the implementation at the operational level. It is a complete and clear document with its strengths and weaknesses (Table 5). However, this pilot study provides complementary practical information raising problems encountered with the traps in the field and suggesting solutions to address these problems, and a comparison of estimated and actual costs and workload. These findings will contribute to improve the guidelines, which must be adapted to the local context (as suggested in the guidelines), taking into account salary and investment costs, available expertise and the required workload in the preparatory phase. Initial training in identification, larval search, trap positioning, and continuing support and quality checks are essential in making the surveillance programme successful. Finally, a favourable legal context to implement the necessary vector control measures should be created in Belgium, by registering appropriate insecticides for mosquito control, in accordance with EU regulation.

**Table 5:** Comparison of the strengths and weaknesses of the ECDC guidelines

**Strengths**	**Weaknesses**
• Easy development of surveillance strategies based on the three scenarios. Complete and clear information on the scenario’s and procedures to be followed	• Some specific issues such as the problems that may occur with the traps in the field are not addressed
• Useful summary and checklist boxes, with appropriate explanatory tables and figures	• Illustration of the main characteristics of larvae and adult mosquitoes used in the identification keys are not provided
• Operational implementation adaptable to local context	• Number, degree and skills of people needed to be involved in the surveillance are not specified
• Cost and workload estimation is provided	• Preparatory costs and workload are not included in the estimation

## Competing interests

ECDC coordinated the pilot project (LMR) and a member of VBORNET provided technical support during the study implementation (FS). They did not interfere with the evaluation relative to usefulness and applicability of the guidelines recommendations, which was performed by the ITM team only. Thus, recommendations and evaluations are performed by independent bodies.

## Authors’ contributions

ID coordinated the study and drafted the manuscript. ID and CS designed the study, carried out the fieldwork and identifications, analysed the results, and evaluated the feasibility of the operational implementation of the ECDC guidelines. MC participated in the design of the study, its coordination and the evaluation. FS provided advice during the study design and trained ID and CS for mosquito identification and field surveillance implementation. LMR coordinated the ECDC pilot project. All authors read and approved the final manuscript.

## Supplementary Material

Additional file 1 Coordinates of the surveyed points of entry (PoE) and the two areas colonised by invasive mosquito species (IMS). Click here for file

Additional file 2 Entity relationship diagram of the MS Access database used in the ExoSurv project. Click here for file

Additional file 3 Number of adult (MMLP trap) or larval mosquitoes captured per species at each type of PoE. Click here for file

Additional file 4 **Working costs (travel, consumables) and workload estimation of all tasks by procedure based on the actual observed number of sites, km**^**2 **^**and working days (wd) of the ExoSurv project applying the ECDC cost and workload rates [**[28]**].** Click here for file

Additional file 5 **Detailed costs and workload estimation for field investigations based on the actual observed number of sites and visits, km, km**^**2 **^**and working days of the ExoSurv project applying the ECDC formulas [**[28]**].** Click here for file
