# Supplementary material for: Implementation of surveillance of invasive mosquitoes in Belgium according to the ECDC guidelines
Source: Parasit Vectors. 2014 Apr 26;7:201. doi: 10.1186/1756-3305-7-201 (PMC4021692; doi:10.1186/1756-3305-7-201)
Supplement: Additional file 2 — Entity relationship diagram of the MS Access database used in the ExoSurv project. [file 1756-3305-7-201-S2.pdf]

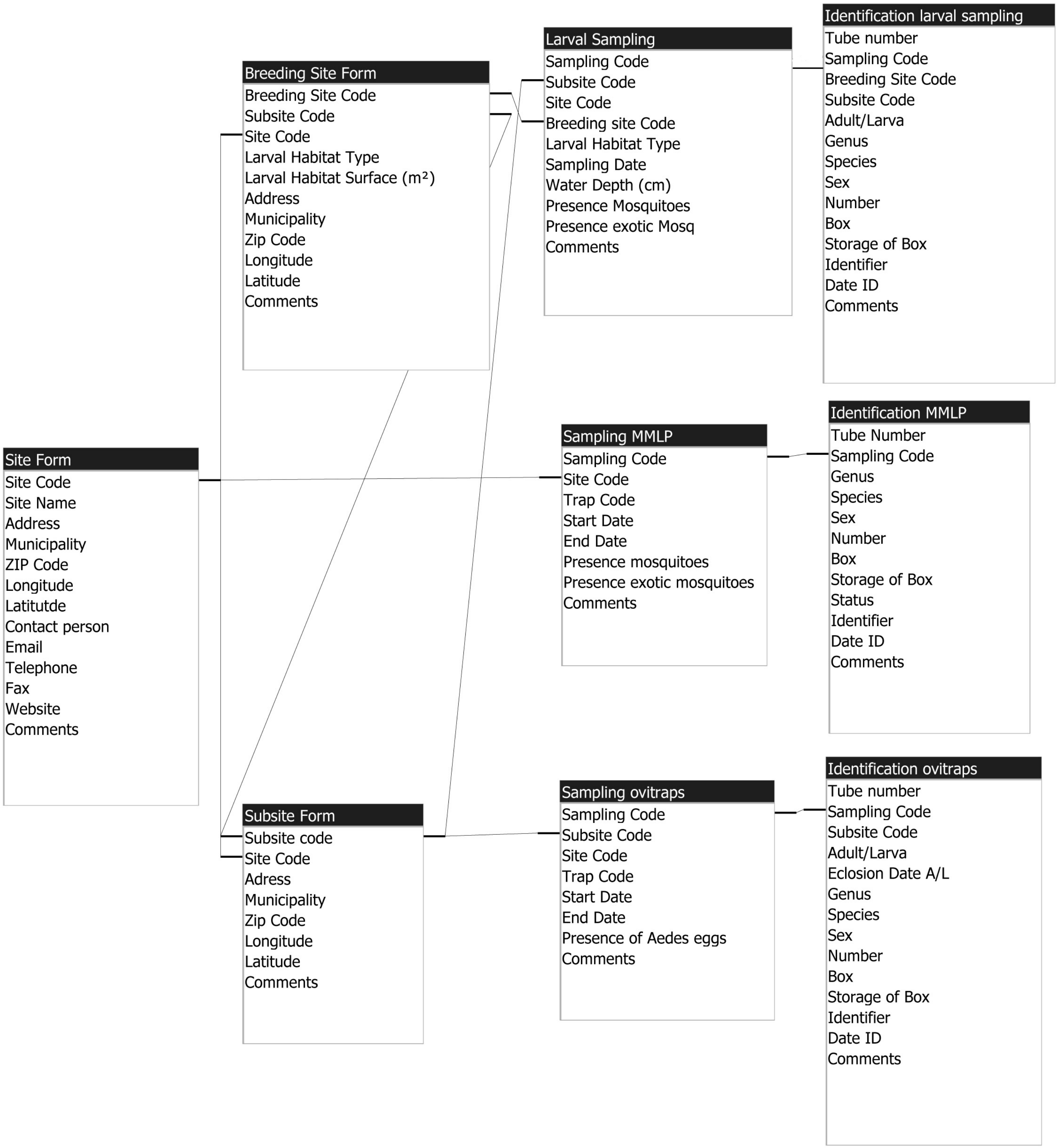
Identification larval sampling
Tube number
Sampling Code
Breeding Site Code
Subsite Code
Adult/Larva
Genus
Species
Sex
Number
Box
Storage of Box
Identifier
Date ID
Comments
Larval Sampling
Sampling Code
Subsite Code
Site Code
Breeding site Code
Larval Habitat Type
Sampling Date
Water Depth (cm)
Presence Mosquitoes
Presence exotic Mosq
Comments
Breeding Site Form
Breeding Site Code
Subsite Code
Site Code
Larval Habitat Type
Larval Habitat Surface (m²)
Address
Municipality
Zip Code
Longitude
Latitude
Comments
Identification MMLP
Tube Number
Sampling Code
Genus
Species
Sex
Number
Box
Storage of Box
Status
Identifier
Date ID
Comments
Sampling MMLP
Sampling Code
Site Code
Trap Code
Start Date
End Date
Presence mosquitoes
Presence exotic mosquitoes
Comments
Site Form
Site Code
Site Name
Address
Municipality
ZIP Code
Longitude
Latitutde
Contact person
Email
Telephone
Fax
Website
Comments
Identification ovitraps
Tube number
Sampling Code
Subsite Code
Adult/Larva
Eclosion Date A/L
Genus
Species
Sex
Number
Box
Storage of Box
Identifier
Date ID
Comments
Sampling ovitraps
Sampling Code
Subsite Code
Site Code
Trap Code
Start Date
End Date
Presence of Aedes eggs
Comments
Subsite Form
Subsite code
Site Code
Adress
Municipality
Zip Code
Longitude
Latitude
Comments
